# Disease-specific health literacy, disease knowledge, and adherence behavior among patients with type 2 diabetes in Taiwan

**DOI:** 10.1186/s12889-018-5972-x

**Published:** 2018-08-24

**Authors:** Jue-Zong Yeh, Chung-jen Wei, Shuen-fu Weng, Cheng-yu Tsai, Jia-hui Shih, Chung-liang Shih, Chiung-hsuan Chiu

**Affiliations:** 1 Department of Pharmacy, Tri-Service General Hospital, Taipei, Taiwan; 2 Department of Public Health, Fu Jen Catholic University, New Taipei City, Taiwan; 3 Division of Endocrinology and Metabolism, Department of Internal Medicine, Taipei Medical University Hospital, Taipei, Taiwan; 4 Division of Endocrinology and Metabolism, Department of Internal Medicine, School of Medicine, College of Medicine, Taipei Medical University, Taipei, Taiwan; 5 School of Health Care Administration, Taipei Medical University, 250 Wu-hsing St., Taipei, Taiwan; 6 Ministry of Health and Welfare, Taipei, Taiwan

**Keywords:** Diabetes-specific health literacy, Diabetes-specific health knowledge, Adherence behavior

## Abstract

**Background:**

To examine the association between health literacy, level of disease knowledge, and adherence behavior among patients with type 2 diabetes.

**Methods:**

A cross-sectional survey study of 1059 Mandarin- and Taiwanese-speaking patients aged 20 years or older with type 2 diabetes was conducted. The demographic profiles of the sample strata were determined by analyzing the Taiwanese National Health Insurance Database. Participants were enrolled and completed questionnaires between April and November of 2015. The patients were assessed using a self-developed questionnaire with high internal consistency (KR-20 = .84).

**Results:**

Construct validity was supported by Confirmatory Factor Analysis. Respondents scored lowest in diet-related knowledge. Health literacy and diabetes knowledge were significantly greater when patients cared for themselves with additional caretaker assistance. Patient age, gender, and educational attainment were associated with adherence behavior.

**Conclusion:**

This study conducted a nation-wide survey of patients with diabetes and the results showed that respondents possessed fairly strong diabetes-specific health literacy and knowledge. However, health literacy shouldn’t be assessed as an isolated concept. Instead, it should be assessed in conjunction with adherence behavior.

## Background

To manage the large population of people with type 2 diabetes, policy makers must focus on understanding the factors that lead to poor control of diabetes. Assessing health literacy is a feasible method of identifying underlying causes of poor diabetes control and finding targets for intervention related to disease knowledge, abilities, and performance of self-care.

Low health literacy of chronic disease patients is associated with high healthcare costs, suboptimal management of illness, and poor health outcomes, especially for diabetes [1]. The International Diabetes Federation (IDF) estimated that the global population of people with diabetes was 415 million in 2015, and this number is expected to rise to 642 million by 2040 [2]. The prevalence of diabetes in Taiwan has increased at an alarming rate from 6.5% in 2002 to 9.2% in 2008 [3, 4]. Diabetes is a major cause of mortality and morbidity because of its complications [5]. Research demonstrates that 20–50% of patients, either intentionally or unintentionally, do not adhere to treatment regimens [6, 7]; this limits the effectiveness of therapies and significantly increases complications [8]. Non-adherence is a major barrier to successful chronic disease management [9] and leads to increased health care costs, hospitalizations, and patient mortality [1, 10, 11].

To address the high risk of poor diabetes management, the Taiwanese government introduced the ‘Diabetes Shared Care Model’ in Yi-lan County in 1996 and implemented it in all other counties by 2002. The Shared Care Model as well as the Diabetes Medical Quality Improvement Project (DMQIP) are funded by the National Health Insurance program to encourage trained medical professionals to utilize standardized teaching materials and care protocols to educate patients with diabetes, as well to promote checking patients’ HbA1c regularly. As of 2015, 42.99% of all patients with diabetes were covered by the DMQIP [12]. 30% of patients covered by DMQIP reached the optimal HbA1c by utilizing collaborative interdisciplinary care with the involvement of physicians, nurses, pharmacists, and official/unofficial social support systems [13].

In addition to external reinforcement, patients must motivate themselves to consume appropriate daily diets, adhere to their prescribed medication regimens, maintain a healthy lifestyle, monitor blood sugar levels, and most importantly maintain these positive behaviors throughout their lifetime despite the inconvenience. Goal-directed adherence behavior is a feasible pathway to appropriate management of diabetes [14]. However, adequate health literacy is the first step in rationalizing self-care behaviors and strengthening the ability to correctly evaluate health-related information. General health literacy is not sufficient, and disease-specific knowledge and reasoning is crucial.

Health literacy is defined by Kutner et al. as ‘the degree to which individuals have the capacity to obtain, process, and understand basic health information and services needed to make appropriate health decisions [15]’. The World Health Organization defines health literacy as ‘the cognitive and social skills which determine the motivation and ability of individuals to gain access to, understand, and use information in ways which promote and maintain good health [16]’. Based on Nutbeam [17], health literacy includes three levels: basic/functional literacy, communicative/interactive literacy, and critical literacy. Schulz and Nakamoto further argue that health literacy develops through the interaction of medical knowledge, experience, comprehension, and reading/numeracy skills [14]. Therefore, the domain of health literacy includes cognitive and social skills that incorporate the abilities required to access, read, comprehend, and analyze information.

Low health literacy is arguably one of the main risk factors for medication and health behavior non-adherence [18]. Individuals with poor health literacy have a heightened risk for poor disease control, as well as an increased risk of complications and prolonged hospitalization [19–21] due to difficulties comprehending and following medical instructions [21, 22]. Poor comprehension of medical instructions is one factor leading to unintentional non-adherence [23]. Nutbeam considers health literacy to be a primary goal of public health education and communication strategies [17]. Due to the fundamental role of health literacy, disease knowledge is required to achieve adequate control and prevent adverse outcomes [24]. Research has demonstrated that knowledge of medications, diet, exercise, glucose monitoring, and treatment modification are necessary to effectively self-manage diabetes [25, 26]. The study conducted by Kravitz et al. showed that diabetes patients had difficulties adhering to all aspects of treatment regimens because different knowledge and skills were required for different aspects of the regimens [27]. Lack of information and deterioration of knowledge also contribute to non-adherence [28, 29].

Adherence to health regimens is a major factor in achieving optimal diabetes outcomes. Many studies indicate a strong positive association between adherence behavior and disease outcomes, and as many as 200 factors have been hypothesized to impact adherence [30]. However, the existing literature on the impact of disease knowledge and health literacy on adherence still remains inconclusive; many studies provide mixed or conflicting results on this topic [9].

Modulated by both external (health care professionals, government, etc.) and internal (the patients themselves) influences, does health literacy and disease knowledge correlate with patients’ health behaviors? This study aims to examine the association between disease-specific health literacy, disease knowledge, and adherence behavior of patients with type 2 diabetes in Taiwan.

## Methods

### Developing the tool

**Fig. 1:**
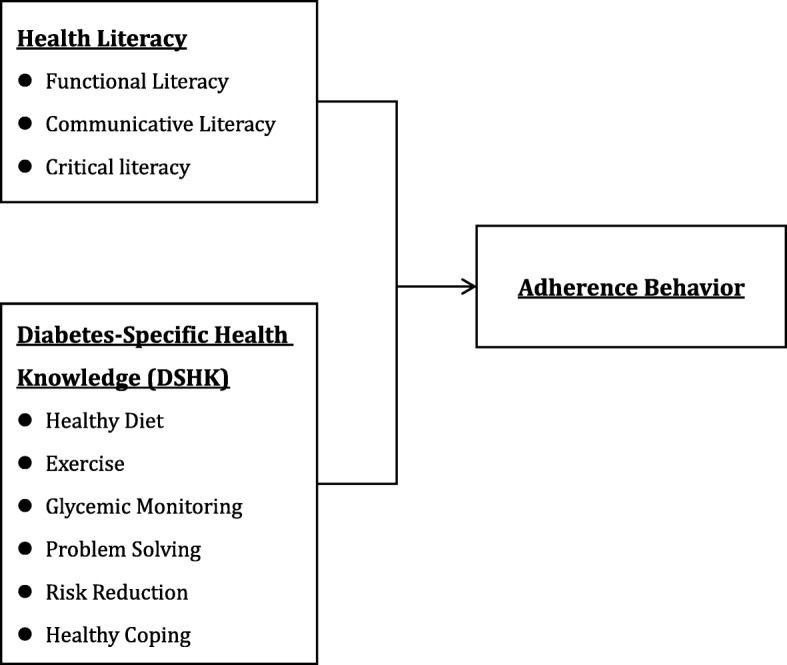
Conceptual framework

Currently available health literacy assessments such as the Test of Functional Health Literacy in Adults (TOFHLA), Wide Range Achievement Test (WRAT), and Rapid Estimate of Adult Literacy in Medicine (REALM) utilize a format that assesses literacy through reading questions and Cloze tests [31–34]. The utility of a health literacy assessment tool is determined by its ability to measure patients’ understanding in the context of their own health and their ability to communicate with their health care providers. The instrument should assess whether patients are able to absorb and comprehend the meaning of health information in the context in which it is encountered. Therefore, based on the relevant literature regarding knowledge of disease and self-care, we devised an instrument with items aligned with the conceptual framework (shown in Fig. 1).

To measure health literacy, the scale items were constructed to directly reflect Nutbeam’s definitions of functional, communicative, and critical health literacy [17]. The items were developed through literature review, patient focus groups, patient education materials from the Taiwanese Association of Diabetic Education (TADE), and panel discussions with various experts (including endocrinologists, public health researchers, pharmacists, nurses, dietitians, health educators, and social science researchers) working in related fields. For functional health literacy, two items were developed to assess the skills involved in reading and understanding basic instructions or handouts from hospitals. Communicative health literacy was assessed using five items addressing the skills needed to extract information, to comprehend and communicate, and to apply new information to changing circumstances. Finally, critical health literacy was assessed using five items that evaluated the participants’ ability to process quantitative information and use it to make further decisions. All twelve items were evaluated using multiple-choice questions with three possible answer choices. One point was given for each item if the respondent answered correctly.

Diabetes-specific knowledge was measured with twenty-five true/false questions addressing six aspects of diabetes knowledge: healthy diet, exercise, glycemic monitoring, problem solving, risk reduction, and healthy coping. Items were developed based on existing diabetes knowledge scales [19, 21, 24, 35–37] and Taiwanese patient education materials [38]. For each item, patients were given three choices: ‘true’, ‘false’, or ‘don’t know’. One point was given for each item if the respondent answered correctly.

Participants’ adherence behavior with self-care recommendations was measured with ten items encompassing dietary adherence, glycemic monitoring behavior, medication adherence, and exercise adherence. These items used a five-point Likert scale to measure the frequency of these adherence behaviors. These items were developed based on existing compliance/adherence questionnaires [39–41].

For the last section of the questionnaire, demographic and illness-related data were collected. These items included: gender, age, educational attainment, history of diabetes, self-reported HbA1c level, caregiver identity, insulin use, type of medical specialist visited, residential region, and accreditation level of the health care facility where the respondent receives care.

### Validity

In order to ensure face validity, the questionnaire was constructed by experts using the three-run Delphi method. A group of fourteen experts was invited to assess the instrument. This group was composed of five physicians, two associate professors with PhD degree, one MD/PhD holder, three nurses, two pharmacists, and two dietitians. Items were selected based on the experts’ scoring of items: 1 (should remove from the questionnaire), 2 (should keep the item after suggested revisions), and 3 (should keep the item). Items that scored an average of 2 or higher were kept.

Further confirmatory factor analysis was conducted to check construct validity. For reliability, a pilot test was conducted (*n* = 90) in a medical center, a regional hospital, and a district hospital. The reliability of the health literacy and disease knowledge assessments were .894 and .792 respectively based on the Kuder-Richardson Formula 20 [42].

### Setting and study participants

To obtain generalizability to the total population of people with diabetes in Taiwan, probability proportional to size sampling (PPS) was used to determine the number of cases that must be collected based on the clustering criteria of health care facility accreditation level and region of Taiwan. The Taiwanese National Health Insurance database was used to identify the total number and distribution of patients with diabetes by the given criteria. The proportion of samples to be collected from medical centers, regional hospitals, district hospitals, and community clinics in the Northern, Central, Southern, and Eastern (including outlying islands) regions of Taiwan was determined based on this information. From the total population of 1.4 million people with diabetes in Taiwan, 1059 valid responses were collected proportionally from the different regions and health care facility accreditation levels. Based on the equation $n=\frac{\frac{z^2\bullet p\left(1-p\right)}{e^2}}{1+\left(\frac{z^2\bullet p\left(1-p\right)}{e^2N}\right)}$, this maintains a 95% level of confidence with a given sampling error of ±3%. The research team obtained consent for data collection at eight medical centers (yielding 21.9% of total samples), 24 regional hospitals (29.7%), 25 district hospitals (15.6%), and 134 community clinics (32.9%). The conditions requested by these facilities (e.g. very conservative and unobtrusive recruitment strategies) and widespread mistrust of strangers requesting personal information caused by the high prevalence of sophisticated identity theft scams in Taiwan led to a predicted response rate of 18–25% [43]. Therefore, 5600 total possible participants were approached at the selected facilities on a convenience basis. Coordinators monitored data collection to ensure response quality as well as proportional representation by health care facility accreditation level and region.

Data were collected in face-to-face interviews from April to August of 2015. Inclusion criteria required patients with type 2 diabetes to be older than 20 and able to speak either Mandarin or Taiwanese. All interviewers were trained to ensure uniform interviewing skills before data collection. Specific interviewing skills included: approaching and inviting participants, disclosure of the purposes of the study, how to correctly read the questionnaire items in Mandarin and Taiwanese, how to assist interviewees during data collection without revealing answers, how to conclude the interview, and so on. Interviewers invited patients with diabetes to participate in the study outside of endocrinologists’, family physicians’, and general internal medicine physicians’ practice settings. After explaining the purpose and procedure of the study, written and oral consent were obtained from the participants. To facilitate understanding of the study and consent process, the consent form was written at a primary school level. Additionally, interviewers fully explained the interviewees’ rights as a research participant before attempting to obtain consent. Respondents were given an educational diabetes handout after the survey. A total of 1059 samples were collected after contacting 5530 eligible patients with type 2 diabetes which yielded a 19.15% response rate. All data were treated and presented confidentially. This study was conducted with the approval of the Joint Institutional Review Board at Taipei Medical University (TMU-JIRB: 201402043).

### Statistical analysis

Confirmatory factor analysis was used to confirm the instrument’s construct validity. Descriptive statistics were reported for demographic variables, health literacy, and diabetes knowledge. After ensuring that the study variables followed a normal distribution, hierarchical linear modeling was used to examine the relationship between disease-specific health literacy, disease knowledge, and adherence behavior by health care facility accreditation level. Data were analyzed using the Statistic Package for the Social Sciences (SPSS) version 22.0, Statistical Analysis System (SAS) version 9.4, and Linear Structural Relationships (LISERL) version 9.2.

## Results

### Construct validity

**Fig. 2:**
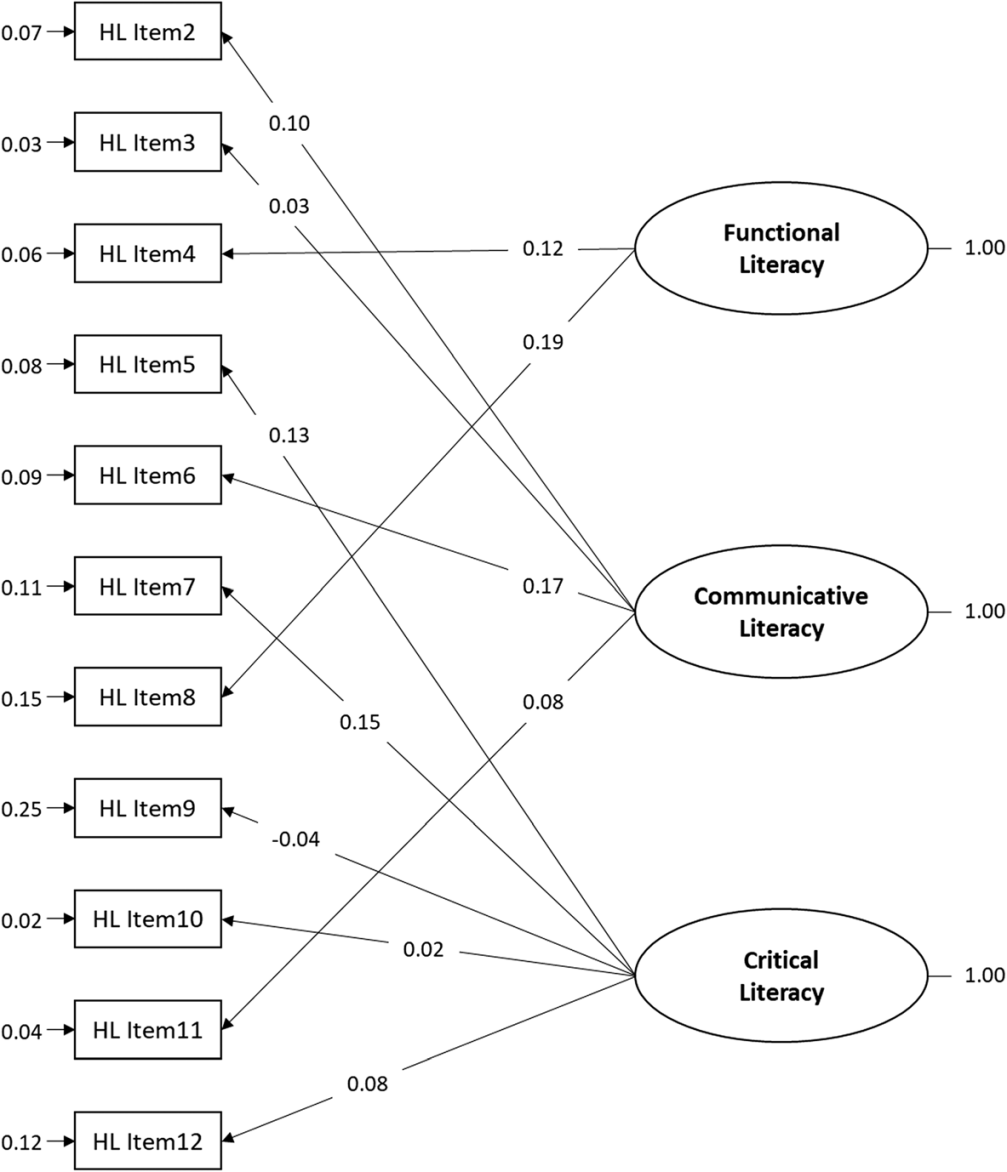
Confirmatory factor analysis of the 3 dimensions of health literacy. (*n* = 1059)

**Fig. 3:**
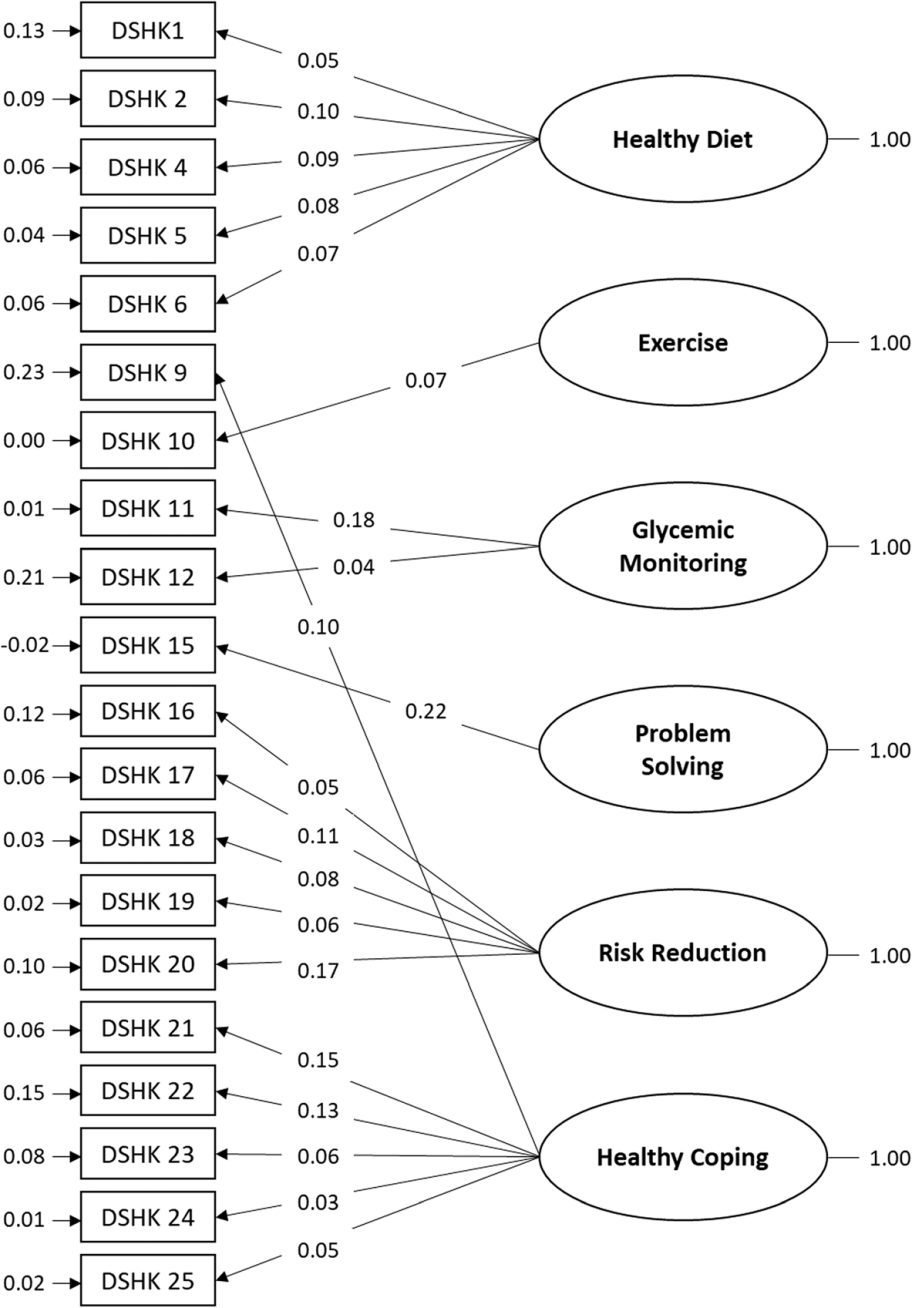
Confirmatory factor analysis of the 6 dimensions of diabetes-specific health knowledge (DSHK). (*n* = 1059)

To confirm the construct validity, confirmatory factor analysis was used to measure items’ factorial structure with LISREL 9.2. Based on the model fit index, eleven items were deleted to ensure appropriate fit and maintain construct integrity regarding the participant’s literacy, knowledge, or behaviors (Table 1). Following construct validity analysis, this instrument retained 11 items in health literacy, 20 items in diabetes knowledge, and 5 items in behaviors. The coefficients of confirmatory factor analysis are shown in Figs. 2 and 3.

**Table 1:** Model fit statistics

	Health Literacy	Diabetes-Specific Health Knowledge	Adherence Behavior	Fitting Index
Overall Model Fit				
Chi-square	100.23	580.32	44.35	Smaller is better
RMSEA	0.03	0.05	0.08	< 0.05
Absolute Fit				
GFI	0.98	0.95	0.98	> 0.9
AGFI	0.97	0.93	0.95	> 0.9
Comparative Fit				
NNFI	0.85	0.67	0.81	> 0.9
CFI	0.89	0.73	0.91	> 0.9
ECVI	0.14	0.62	0.06	Smaller is better
Parsimonious Fit				
PNFI	0.62	0.55	0.45	> 0.5
PGFI	0.61	0.70	0.33	> 0.5

Health literacy includes functional literacy, communicative literacy, and critical literacy

Diabetes-specific health knowledge includes healthy diet, exercise, glycemic monitoring, problem solving, risk reduction, and healthy coping

### Descriptive statistics

A total of 1059 patients who visited hospital medical departments or community clinics between April and November of 2015 were surveyed. The mean age of the patients was 57 years (SD = 10.79, range 20–88) and the mean time since the diagnosis of diabetes was 8 years. 50.9% the patients were male and 76.4% of patients had received at least a high school diploma. The mean HbA1c of patients was 7.45% (SD = 1.25, range 4–14.7). Most patients cared for themselves without an additional caretaker (57.7%), didn’t use insulin (84.9%), and sought care at the endocrinology & metabolism department (61.9%). Most patients reported no smoking (63.5%), no drinking (51.8%), and no chewing betel nut (a stimulant common in parts of Asia) (83.9%).

The patients’ mean score was 1.67 (SD = 0.55, range 0–2) for functional literacy, 3.69 (0.62, 0–4) for communicative literacy, 4.07 (0.83, 1–5) for critical literacy, and 9.44 (1.40, 3–11) for total health literacy. The patients’ mean score for diabetes-related knowledge topics was 3.86 (0.74, 0–5) for healthy diet, 0.99 (0.10, 0–1) for exercise, 1.26 (0.52, 0–2) for glycemic monitoring, 0.97 (0.17, 0–1) for problem solving, 4.57 (0.71, 0–5) for risk reduction, 5.14 (0.92, 1–6) for healthy coping, and 16.79 (1.98, 8–20) for total diabetes knowledge. Patients’ adherence behavior scores averaged 15.03 (4.32, 0–25).

### Bivariate analysis

The t-test and ANOVA were used to examine the association between patients’ disease-specific health literacy, disease knowledge, and adherence behavior. The results showed that there were significant differences based on patient demography (Tables 2 and 3).

**Table 2:** Health literacy and diabetes-specific health knowledge by demographics and health factors (*N* = 1059)

		Health Literacy (0~ 11)			Diabetes-Specific Health Knowledge (0~ 20)					
	N(%)	FUN	COM	CRI	DIET	EXER	MONI	SOLV	RISK	COPING
	Range	(0–2)	(0–4)	(0–5)	(0–5)	(0–1)	(0–2)	(0–1)	(0–5)	(0–6)
Age (years)										
20–29	14(1.3)	1.86(0.36)	4.00(0.00)	4.14(0.53)	3.86(0.36)	1.00(0.00)	1.29(0.83)	1.00(0.00)	4.79(0.58)	5.14(0.66)
30–39	53(5.0)	1.64(0.56)	3.89(0.38)	3.98(0.75)	3.77(0.72)	1.00(0.00)	1.45(0.54)	1.00(0.00)	4.55(0.89)	5.26(0.88)
40–49	172(16.3)	1.72(0.52)	3.66(0.60)	4.08(0.84)	3.83(0.72)	0.99(0.08)	1.24(0.51)	0.99(0.08)	4.53(0.69)	5.31(0.84)
50–59	392(37.1)	1.70(0.54)	3.70(0.62)	4.14(0.82)	3.93(0.70)	0.99(0.09)	1.27(0.54)	0.96(0.19)	4.63(0.69)	5.11(0.93)
Over 60	426(40.3)	1.60(0.59)	3.66(0.66)	4.00(0.86)	3.81(0.79)	0.99(0.12)	1.24(0.50)	0.97(0.18)	4.54(0.72)	5.08(0.94)
*p*-value		0.010*	0.005**	0.126	0.032*	0.455	0.250	0.153	0.366	0.005**
HbA1C (%)										
Under 7.0	407(38.4)	1.70(0.52)	3.72(0.64)	3.99(0.80)	3.84(0.69)	0.99(0.70)	1.31(0.53)	0.98(0.14)	4.57(0.72)	5.21(0.86)
Over 7.1	652(61.6)	1.65(0.58)	3.68(0.60)	4.12(0.84)	3.87(0.77)	0.99(0.11)	1.23(0.52)	0.97(0.18)	4.57(0.71)	5.10(0.95)
*p*-value		0.159	0.327	0.016*	0.483	0.184	0.016*	0.154	0.922	0.042*
Gender										
Male	539(50.9)	1.64(0.56)	3.67(0.64)	4.12(0.83)	3.88(0.75)	0.99(0.10)	1.27(0.52)	0.97(1.70)	4.61(0.73)	5.17(0.89)
Female	520(49.1)	1.69(0.55)	3.72(0.58)	4.02(0.83)	3.83(0.73)	0.99(0.10)	1.25(0.53)	0.97(1.62)	4.53(0.69)	5.10(0.95)
*p*-value		0.087	0.250	0.051	0.307	0.972	0.295	0.753	0.062	0.133
Educational Attainment										
Primary School or Lower	251(23.7)	1.63(0.59)	3.57(0.72)	4.02(0.84)	3.72(0.87)	0.99(0.12)	1.23(0.49)	0.97(0.18)	4.43(0.81)	4.85(1.05)
Junior high	185(17.5)	1.65(0.58)	3.76(0.54)	4.13(0.81)	4.06(0.69)	0.99(0.07)	1.36(0.53)	0.97(0.17)	4.59(0.75)	5.26(0.86)
Senior high	364(34.4)	1.68(0.54)	3.70(0.66)	4.11(0.83)	3.88(0.67)	0.99(0.10)	1.22(0.51)	0.99(0.12)	4.62(0.63)	5.16(0.96)
College or Higher	259(24.5)	1.69(0.53)	3.72(0.54)	3.98(0.84)	3.76(0.76)	0.99(0.11)	1.26(0.55)	0.96(0.19)	4.59(0.72)	5.25(0.84)
*p*-value		0.523	0.018*	0.124	<.001***	0.810	0.017*	0.290	0.007**	<.001***
Patient Caregiver										
Self	611(57.7)	1.62(0.59)	3.66(0.64)	3.98(0.85)	3.80(0.75)	0.99(0.08)	1.20(0.50)	0.97(0.17)	4.55(0.72)	5.12(0.91)
Others	98(9.3)	1.64(0.57)	3.59(0.80)	4.11(0.83)	3.78(0.81)	0.98(0.13)	1.18(0.47)	0.96(0.19)	4.63(0.65)	5.07(1.14)
Self + Others	350(33.1)	1.77(0.47)	3.79(0.52)	4.19(0.79)	3.92(0.75)	0.98(0.13)	1.37(0.55)	0.98(0.14)	4.56(0.75)	5.14(0.97)
*p*-value		<.001***	0.006**	0.001**	0.046*	0.381	<.001***	0.475	0.533	0.815
Specialty Visited										
Endocrinology & Metabolism	654(61.9)	1.64(0.58)	3.70(0.61)	4.05(0.83)	3.80(0.78)	0.99(0.10)	1.29(0.55)	0.97(0.17)	4.58(0.71)	5.12(0.92)
Others	402(38.1)	1.72(0.51)	3.68(0.63)	4.11(0.82)	3.95(0.65)	0.99(0.09)	1.21(0.48)	0.97(0.16)	4.56(0.72)	5.16(0.92)
*p*-value		0.033*	0.551	0.238	0.001**	0.598	0.007**	0.873	0.598	0.451
Insulin Use										
Yes	160(15.1)	1.56(0.60)	3.71(0.63)	3.99(0.79)	3.82(0.79)	0.99(0.11)	1.35(0.50)	0.95(0.22)	4.58(0.76)	5.14(0.91)
No	899(84.9)	1.69(0.55)	3.69(0.61)	4.09(0.73)	3.86(0.73)	0.99(0.09)	1.25(0.53)	0.98(0.15)	4.57(0.70)	5.14(0.92)
*p*-value		0.016*	0.745	0.183	0.494	0.665	0.022*	0.159	0.862	0.982
Health Care Facility Accreditation Level										
Medical Center	232(21.9)	1.56(0.62)	3.69(0.63)	3.90(0.87)	3.59(0.80)	0.99(0.09)	1.30(0.55)	0.96(0.19)	4.47(0.88)	5.20(0.83)
Regional Hospital	314(29.7)	1.67(0.57)	3.73(0.62)	4.13(0.86)	4.02(0.67)	0.99(0.08)	1.31(0.53)	0.98(0.14)	4.74(0.49)	5.29(0.83)
District Hospital	165(15.6)	1.68(0.55)	3.71(0.53)	4.04(0.71)	3.76(0.80)	0.98(0.13)	1.24(0.58)	0.96(0.19)	4.55(0.63)	4.64(1.11)
Community Clinic	348(32.9)	1.74(0.49)	3.66(0.64)	4.14(0.81)	3.93(0.67)	0.99(0.09)	1.21(0.46)	0.97(0.16)	4.50(0.77)	5.19(0.88)
*p*-value		0.004**	0.586	0.006**	<.001***	0.637	0.073	0.536	<.001***	<.001***
Residential Region										
Northern	466(44.0)	1.67(0.57)	3.67(0.65)	4.16(0.89)	3.89(0.82)	0.99(0.08)	1.21(0.54)	0.97(0.16)	4.58(0.73)	5.14(0.98)
Central	251(23.7)	1.78(0.47)	3.83(0.42)	4.20(0.76)	3.83(0.59)	0.98(0.14)	1.27(0.45)	0.96(0.19)	4.53(0.67)	5.04(0.90)
Southern	299(28.2)	1.56(0.58)	3.62(0.67)	3.85(0.75)	3.84(0.71)	1.00(0.06)	1.32(0.54)	0.97(0.16)	4.62(0.71)	5.27(0.82)
Eastern & Outlying Islands	43(4.1)	1.91(0.29)	3.83(0.49)	3.91(0.61)	3.67(0.78)	0.98(0.15)	1.37(0.58)	1.00(0.00)	4.47(0.77)	4.72(0.93)
*p*-value		<.001***	<.001***	<.001***	0.222	0.319	0.017*	0.616.	0.321	<.001***

*FUN* functional literacy, *COM* communicative literacy, *CRI* critical literacy, *DIET* healthy diet, *EXER* exercise, *MONI* glycemic monitoring, *SOLV* problem solving, *RISK* risk reduction, *COPING* healthy coping

* *p* < 0.05,** *p* < 0.01,*** *p* < 0.001

**Table 3:** Adherence behavior by demographics and health factors (*N* = 1059)

	Adherence Behaviors						
	N(%)	Dietary Adherence	Body Weight Monitoring	Glycemic Monitoring	Medication Adherence	Exercise Adherence	Total Score
Age (years)							
20–29	14(1.3)	3.36(1.39)	2.93(1.39)	2.71(1.82)	2.64(1.39)	2.69(1.65)	14.77(4.87)
30–39	53(5.0)	2.87(1.42)	2.49(1.54)	2.78(1.46)	3.80(1.16)	2.02(1.59)	13.49(4.61)
40–49	172(16.3)	3.21(1.42)	2.52(1.33)	2.60(1.66)	3.85(0.99)	2.15(1.67)	14.40(4.55)
50–59	392(37.1)	3.41(1.42)	2.53(1.35)	2.53(1.66)	3.96(0.89)	2.43(1.67)	14.80(4.19)
Over 60	426(40.3)	3.50(1.46)	2.60(1.47)	2.68(1.75)	4.04(0.95)	2.84(1.76)	15.64(4.22)
*p*-value		0.017*	0.771	0.685	< 0.001***	< 0.001***	0.001**
HbA1C (%)							
Under 7.0	407(38.4)	3.52(1.37)	2.65(1.38)	2.79(1.66)	3.98(0.91)	2.75(1.72)	15.69(4.05)
Over 7.1	652(61.6)	3.30(1.48)	2.50(1.42)	2.51(1.70)	3.93(1.00)	2.40(1.72)	14.61(4.43)
*p*-value		0.015*	0.091	0.009**	0.371	0.001**	< 0.001***
Gender							
Male	539(50.9)	3.26(1.51)	2.46(1.41)	2.57(1.67)	3.92(0.95)	2.48(1.76)	14.67(4.36)
Female	520(49.1)	3.51(1.36)	2.67(1.39)	2.67(1.72)	3.98(0.98)	2.60(1.69)	15.41(4.25)
*p*-value		0.006**	0.013*	0.334	0.298	0.253	0.007**
Educational Attainment							
Primary school or lower	251(23.7)	3.57(1.50)	2.44(1.48)	2.56(1.79)	4.00(1.03)	2.66(1.83)	15.19(4.50)
Junior High	185(17.5)	3.33(1.40)	2.37(1.28)	2.41(1.58)	3.93(0.87)	2.17(1.76)	14.23(4.00)
Senior High	364(34.4)	3.31(1.39)	2.60(1.36)	2.65(1.67)	4.03(0.84)	2.48(1.70)	15.08(4.41)
College or Higher	259(24.5)	3.35(1.48)	2.76(1.44)	2.78(1.69)	3.81(1.12)	2.75(1.58)	15.39(4.21)
*p*-value		0.146	0.010*	0.148	0.041*	0.003**	0.042*
Patient Caregiver							
Self	611(57.7)	3.28(1.49)	2.48(1.41)	2.43(1.77)	3.97(1.01)	2.65(1.73)	14.78(4.30)
Others	98(9.3)	3.38(1.54)	2.68(1.47)	2.57(1.72)	3.80(1.12)	2.07(1.82)	14.45(4.44)
Self + Others	350(33.1)	3.57(1.30)	2.66(1.37)	2.96(1.47)	3.95(0.84)	2.47(1.67)	15.62(4.27)
*p*-value		0.012*	0.113	< 0.001***	0.289	0.006**	0.008**
Specialty Visited							
Endocrinology & Metabolism	654(61.9)	3.49(1.40)	2.68(1.43)	2.77(1.67)	3.94(0.97)	2.64(1.70)	15.49(4.24)
Others	402(38.1)	3.20(1.50)	2.37(1.34)	2.37(1.71)	3.97(0.97)	2.37(1.76)	14.26(4.37)
*p*-value		0.002**	< 0.001***	< 0.001***	0.698	0.015*	< 0.001***
Insulin Use							
Yes	160(15.1)	3.29(1.40)	2.55(1.52)	3.08(1.60)	3.76(1.08)	2.34(1.75)	14.50(4.74)
No	899(84.9)	3.40(1.45)	2.56(1.38)	2.54(1.69)	3.98(0.94)	2.57(1.72)	15.11(4.25)
*p*-value		0.357	0.923	< 0.001***	0.028*	0.113	0.136
Health Care Facility Accreditation Level							
Medical Center	232(21.9)	2.95(1.68)	2.54(1.60)	2.43(1.81)	3.88(1.08)	2.71(1.69)	14.55(4.74)
Regional Hospital	314(29.7)	3.52(1.26)	2.74(1.33)	2.82(1.58)	3.89(1.03)	2.61(1.74)	15.53(4.11)
District Hospital	165(15.6)	3.68(1.32)	2.60(1.43)	2.74(1.68)	3.94(0.90)	2.52(1.69)	15.39(4.44)
Community Clinic	348(32.9)	3.41(1.42)	2.40(1.30)	2.51(1.69)	4.05(0.84)	2.37(1.75)	14.72(4.10)
*p*-value		< 0.001***	0.022*	0.025*	0.136	0.110	0.022*
Residential Region							
Northern	466(44.0)	3.09(1.45)	2.60(1.45)	2.45(1.66)	3.96(0.98)	2.28(1.71)	14.38(4.25)
Central	251(23.7)	4.06(1.19)	2.90(1.35)	2.95(1.73)	4.13(0.78)	3.00(1.58)	16.93(4.24)
Southern	299(28.2)	3.23(1.46)	2.29(1.30)	2.60(1.65)	3.81(1.05)	2.56(1.77)	14.55(4.08)
Eastern & Outlying Islands	43(4.1)	3.69(1.26)	2.12(1.40)	2.63(1.80)	3.74(1.00)	2.40(1.92)	14.50(4.11)
*p*-value		< 0.001***	< 0.001***	0.002**	0.001**	< 0.001***	< 0.001***

Total score = dietary adherence + body weight monitoring + glycemic monitoring + medication adherence + exercise adherence

* *p* < 0.05,** *p* < 0.01,*** *p* < 0.001

The results showed that functional literacy varied significantly by age, patient caregiver identity, insulin use, health care facility accreditation level, and residential region. Communicative literacy varied significantly by patient caregiver identity and residential region. Critical literacy varied significantly by HbA1c, patient caregiver identity, health care facility accreditation level, and residential region.

For diabetes-specific knowledge, knowledge of a healthy diet varied significantly by educational attainment, the department or type of specialist the patient visited, and health care facility accreditation level. Knowledge of glycemic monitoring varied significantly by HbA1c, educational attainment, patient caregiver identity, the department or type of specialist the patient visited, and residential region. Knowledge of risk reduction varied significantly by educational attainment and health care facility accreditation level. Knowledge of healthy coping varied significantly by HbA1c, educational attainment, health care facility accreditation level, and residential region.

For adherence behavior (Table 3), dietary adherence varied significantly by age, HbA1c, gender, patient caregiver identity, the department or type of specialist the patient visited, health care facility accreditation level, and residential region. Medication adherence behavior varied significantly by age and residential region. Exercise adherence varied significantly by age, HbA1c, educational attainment, patient caregiver identity, the department or type of specialist the patient visited, and residential region. Overall adherence behavior (in total) varied significantly by age, HbA1c, gender, educational attainment, patient caregiver identity, the department or type of specialist the patient visited, health care facility accreditation level, and residential region.

### Pearson correlation

In the correlation analysis (Table 4), we addressed each dimension of disease-specific health literacy, diabetes knowledge, and adherence behavior to understand the relationships between the variables. The results showed that functional literacy, communicative literacy, and critical literacy are significantly positively correlated with each other. The dimensions of diabetes knowledge are significantly positively correlated with each other as well. For diabetes knowledge, the dimensions of healthy diet, glycemic monitoring, problem solving, risk reduction, and healthy coping show significant positive correlation with functional literacy and communicative literacy. Healthy diet, glycemic monitoring, problem solving, and healthy coping show significant positive correlation with critical literacy. Exercise knowledge is significantly negatively correlated with adherence behavior.

**Table 4:** Pearson correlations between sub-dimensions of health literacy or diabetes-specific health knowledge and adherence behavior

	FUN	COM	CRI	DIET	EXER	MONI	SOLV	RISK	COPING
COM	.26**	–							
CRI	.21**	.22**	–						
DIET	.15**	.14**	.27**	–					
EXER	−.01	.05	.02	.11**	–				
MONI	.17**	.10**	.08*	.14**	.03	–			
SOLV	.08*	.13**	.07*	.14**	.10**	.03	–		
RISK	.07*	.14**	.03	.35**	.07*	.10**	.20**	–	
COPING	.09**	.16**	.11**	.30**	.07*	.17**	.12**	.33**	–
TOTAL	.02	−.03	−.05	−.04	−.12**	.05	−.03	.03	−.01

*FUN* functional literacy, *COM* communicative literacy, *CRI* critical literacy, *DIET* healthy diet, *EXER* exercise, *MONI* glycemic monitoring, *SOLV* problem solving, *RISK* risks reduction, *COPING* healthy coping, *TOTAL* total adherence behavior score

* *p* < 0.05,** *p* < 0.01,*** *p* < 0.001

### Regression

Hierarchical linear modeling was used to explore the relationship between the control variables (patients’ age, gender, educational attainment, residential region, patient caregiver identity, the department or type of specialist the patient visited, and insulin use) and the dependent variables (health literacy, diabetes knowledge, and adherence behavior) by health care facility accreditation level in order to find the appropriate framework for this study (Table 5).

**Table 5:** Hierarchical liner modeling of the relationship of health literacy and diabetes-specific health knowledge with adherence behavior

Independent	Dependent	Health Literacy ^a^	Diabetes-Specific Health Knowledge^b^	Adherence Behavior	
				Model 1^c^	Model 2^d^
		β coefficient	β coefficient	β coefficient	β coefficient
Age		−0.01*	−0.01	0.08***	0.08***
Gender (Female)					
Male		−0.02	0.12	−0.75**	−0.83**
Educational Attainment (Primary school or less)					
Junior High		0.25	0.68***	0.03	0.28
Senior High		0.18	0.41*	1.13**	1.43**
College Or Higher		0.20	0.35	1.84***	2.01***
Residential Area (Northern)					
Central		0.28*	−0.12	2.43***	2.20***
Southern		−0.46***	0.20	0.15	−0.09
Eastern & Outer Islands		0.14	−0.37	0.21	0.79
Patient Caregiver (Self)					
Others		0.20	0.23	−0.46	−0.34
Self + Others		0.50***	0.44***	0.65*	0.57
Specialty Visited (Others)					
Endocrinology & Metabolism		−0.09	−0.28	0.13***	1.22***
Insulin Use (No)					
Yes		−0.16	0.03	−0.02	− 0.21
【Health Literacy】					
Functional Literacy					0.07
Communicative Literacy					−0.40
Critical Literacy					−0.21
【Diabetes Knowledge】					
Healthy Diet					−0.22
Exercise					−4.03*
Glycemic Monitoring					0.39
Problem Solving					0.26
Risks Reduction					0.11
Healthy Coping					−0.004
Random Effect					
Variance of Health Care Facility Level (Residual)		0.014(0.020)	0.211(0.185)	0.182(0.223)	0.135(0.194)
Intra-Class Correlation (ICC)		0.008	0.055	0.011	0.008
*p* Value		0.234	0.128	0.207	0.243

This table reports standardized beta coefficients

Intra-Class Correlation (ICC) = Variance of health care facility level /(Variance of health care facility level + residual)

Regression equations:

^a^health literacy = demographic variables

^b^disease-specific health knowledge = demographic variables

^c^adherence behavior model 1: adherence behavior = demographic variables

^d^adherence behavior model 2: adherence behavior = demographic variables + health literacy + disease-specific knowledge

* *p* < 0.05,** *p* < 0.01,*** *p* < 0.001

In order to assess the possible moderating effect of demographic variables, a two-step model was used as shown in Table 5. Model 1 was used to identify a possible causal relationship between demographic variables and respondents’ adherence behavior; model 2 included demographic variables, health literacy, and disease knowledge as independent variables to further assess their impact on adherence behavior. Since there was no significant change in beta, no moderating effect of demographic variables was found in this case.

Patients’ age, residential region, and patient caregiver identity were associated with their health literacy. Health literacy declined with increasing age, and the health literacy of patients living in Central Taiwan was better than that of patients living in Northern Taiwan, which was in turn better than that of patients living in Southern Taiwan. Patients’ educational attainment and patient caregiver identity were associated with their diabetes knowledge. Patients’ residential region and caregiver identity were associated with their health literacy and diabetes knowledge. Furthermore, the diabetes knowledge of patients living in Northern Taiwan was better than that of patients living in Eastern Taiwan including the outlying islands. Health literacy and diabetes knowledge were significantly greater when patients cared for themselves with additional caretaker assistance. Patients’ age, gender, educational attainment, residential region, and the department or type of specialist the patient visited were associated with their adherence behavior. Finally, diabetes-specific knowledge of exercise was negatively associated with adherence behavior.

## Discussion

### Representativeness of samples

This study intended to collect nation-wide data that provided a fair representation of the national population of people with diabetes. Compared to the demographic statistics of the population, this study had a similar gender distribution: 50.9% males in the sample compared to 50.7% males in the total population of people with diabetes. However, the study had younger respondents (40.3% were older than 60 years) compared to the age composition of the Taiwanese population of people with diabetes (58.1% were older than 60 years). This may be because this study only surveyed respondents who were able to comprehend and respond to the assessment, which may exclude a larger proportion of older individuals. Of those approached but not included in the sample, 54 did not meet inclusion criteria, 4423 refused to participate or had their caretaker refuse participation, and 153 dropped out after beginning the questionnaire. The reasons why individuals dropped out were not collected.

To further assess the representativeness of the sample in this study, the sample was compared with the National Health Interview Survey (NHIS) which included 827 samples stratified from all inhabitants of Taiwan. The percentage of respondents in this study sample who reported not performing glycemic monitoring during the past month was 8.33, 14.59, and 16.95% in the 25–39, 40–64, and ≥65 age groups respectively and 53.69, 60.34, and 60.03% respectively in the NHIS sample [44]. Therefore, this study sample reported more frequent glycemic self-examination behavior in all age groups. While the NHIS was conducted in community settings, this study sample was collected in health care facility settings; this may explain why the respondents in this study sample reported better adherence behavior than average patients with diabetes.

### Instrument

Most health literacy instruments are intended to measure general health literacy [28–31]. Relatively few instruments measure diabetes-specific health literacy [35, 45, 46]. Compared to other diabetes-specific health literacy instruments, this instrument focuses more on the respondents’ knowledge and judgment in identifying appropriate choices. This tool’s focus, good reliability, and validity make it ideal for measuring the deficiencies of knowledge or judgment relating to appropriate self-care and disease management, especially as health literacy relates to Mandarin or Taiwanese language characteristics.

In order to comprehensively assess health literacy and knowledge, this instrument included 3 sub-constructs of health literacy and 6 sub-constructs of health knowledge in the original instrument, with only a few items removed for the final version. Based on further examination of the composite of health knowledge items by confirmatory factor analysis, the exercise and problem solving sub-constructs of health knowledge are recommended for modification for future data collection. For example, ‘Regular exercise and good diet help in the control of blood sugar’ in the exercise sub-construct and ‘Patients with DM should have sugar or sweets available at all times in case of low blood sugar’ in the problem solving sub-construct are so similar to standard health education materials that they reached a 97% correct response rate. On the other hand, some items had relatively low correct response rates: 15.8% on ‘One serving of whole grain food (such as rice or noodles) contains 30g of carbohydrates’ in the healthy diet sub-construct and 30.4% on ‘testing urinary sugar is the best way to measure DM patient’s control of the disease’ in the glycemic monitoring construct. The remaining items had correct response rates from 57.4 to 95.2%. Therefore, this instrument included a combination of easy, moderate, and difficult health knowledge items. The composite of health literacy items had correct response rates ranging from 46.2 to 96.7%, confirming the combination of easy, moderate, and difficult items.

### Major findings

This study reported moderate correct response rates in functional and communicative health literacy as well in the exercise and problem solving sub-constructs of health knowledge. Low correct response rates occurred for critical health literacy and the healthy diet sub-construct of health knowledge. Critical health literacy included numeracy skills (ability to use quantitative information) and using this information to make further decisions; the study results revealed respondents had poor numeracy skills. The numeracy items included medication adherence, reading and comprehension of the prescription and drug label, and medication expiration awareness. Though no association between poor numeracy skills and adherence behavior was found in this study, other studies have shown poor numeracy skills to be associated with medication adherence failure and poor health [20, 47, 48]. Most studies conflate the concepts of health literacy and health knowledge [49]. This study separated these two concepts and examined their association with adherence behavior, as it is believed that low health literacy worsens diabetes and other chronic disease health outcomes and is significantly related to educational level, ethnicity, and age [41]. This study shows that women, older adults, and patients at regional- or district-level hospitals have better adherence behavior than men, younger adults, and patients at community clinics and medical center-level hospitals respectively. Patients with mean HbA1c levels below the treatment guidelines’ recommended target value of 7 mg/dL had better adherence behavior than patients who exceeded the recommended target value.

Studies agree that healthy behavioral changes and weight loss can significantly prevent or reduce the risk of type 2 diabetes [50]. Every 1 kg reduction in body weight can reduce the relative risk of diabetes by 16% and the relative risk of mortality by 15% [51]. The literature consistently shows that it is difficult for patients to maintain adequate weight loss and achieve the desired health outcomes through health-related behavioral changes, especially for obese people with diabetes with weight loss difficulties [52–54]. Knowledge does not necessary lead to good health outcomes, especially as related to exercise. Moreover, most respondents utilized walking and other low-intensity activities as their most common daily exercise, but sustained aerobic exercise is more effective in maintaining a healthy body composition [55, 56]. Additionally, aerobic exercise capacity decreases beginning in middle age [57], and the elderly are less likely choose muscle enhancing activities [58]. Therefore, while knowledge acquisition is relatively easy, adherence to medication regimens, diet, and suggested exercise is only loosely connected to knowledge and health literacy.

This study reported a positive association between age and adherence behavior (Tables 2 and 5). Respondents over 60 years old reported higher dietary, medication, and exercise adherence but were found to have lower functional and communicative literacy. A nutrition and health survey conducted in 1999–2000 for people over 65-years old in Taiwan reported that the elderly lack appropriate knowledge of dietary nutrition and often underestimate their daily nutritional requirements [59]. However, people over 60 may have adjusted their dietary behaviors due to increased difficulties chewing and swallowing. The decline of taste, smell, vision, and other sensory function may also lead to diminished appetite [60, 61]. Cognitive decline and reduced social support can also impact the dietary choices of elderly people [62, 63].

Patients who were cared for themselves with assistance from others performed better in health literacy, diabetes-specific knowledge, and adherence behavior than those who cared for themselves without assistance and those whose care was provided exclusively by another individual. Since diabetes is a chronic and silent illness, adherence to rigid dietary and medication intake regimens can easily fail without external reinforcement. Therefore, added social support may benefit patients’ adherence behavior.

Since the Shared Care Model and Diabetes Medical Quality Improvement Project were introduced in Taiwan 20 years ago, health care facilities participating in these programs have been encouraged to integrate the endocrinology & metabolism department staff (including physicians, nurses, and dietitians) into a network to educate patients about self-care after physician visits. Perhaps due to these programs, patients who visited physicians specializing in endocrinology & metabolism reported higher knowledge of healthy diet and glycemic monitoring than patients visiting physicians practicing other specialties.

A key factor in successfully controlling diabetes is the multidisciplinary team of healthcare professionals who are involved in patient care. The results of the First Basal Insulin Evaluation Asia study in 2015 compared the use of insulin therapy and oral hypoglycemic agents, HbA1c levels, and comorbidities in patients with type 2 diabetes in Asian countries. While Taiwan had the highest prevalence of oral hypoglycemic agent usage, baseline HbA1c levels were also the highest among all analyzed countries. The HbA1c reduction success in Taiwan was minimal, with the lowest proportion of patients achieving HbA1c and Fasting Blood Glucose (FBG) targets at 6 months. Patients with diabetes in Taiwan are typically highly resistant to using insulin, and therefore are late in the course of their disease before they are willing to accept insulin therapy. The authors note that low acceptance of insulin therapy in patients with diabetes in Taiwan is associated with physician prescribing behavior and patient decision-making based on disease management [64].

### Limitations

There are three main limitations of this study. Firstly, perhaps due to the inclusion criteria of the sample (respondents able to comprehend and reply), the use of convernience sampling, and the collection setting, this study collected respondents who were younger and had better adherence behavior than average patients with diabetes in Taiwan. Secondly, the Shared Care Model has been operating for decades. As a result, some health care facilities have developed their own care models to educate and instruct patients with diabetes. The results at some of these facilities have been very good, but this study did not identify patients cared for by those outstanding health care facilities. Finally, results might be biased by the cross-sectional design, i.e. without taking the resources expended by individual facilities into account.

## Conclusion

1. This diabetes-specific health literacy assessment developed for Mandarin and Taiwanese-speaking patients is reliable and well-validated.
2. Health literacy and knowledge are the foundation of making appropriate judgments.
3. Adherence behavior is loosely linked to health literacy and knowledge, especially in chronic disease management.

## Abbreviations

DM: Diabetes
DMQIP: Diabetes Medical Quality Improvement Project
DSHK: Disease-specific Health Knowledge
FBG: Fasting Blood Glucose
HbA1c: Glycated hemoglobin
HPA: Health Promotion Administration
IDF: International Diabetes Federation
LISERL: Linear Structural Relationships
NHIS: National Health Interview Survey
PPS: Probability Proportional to Size Sampling
REALM: Rapid Estimate of Adult Literacy in Medicine
SAS: Statistical Analysis System
SPSS: Statistical Product and Service Solutions
TADE: Taiwanese Association of Diabetic Education
TOFHLA: Test of Functional Health Literacy in Adults
WRAT: Wide Range Achievement Test
